# Human Lung Cancer Cells Grown in an *Ex Vivo* 3D Lung Model Produce Matrix Metalloproteinases Not Produced in 2D Culture

**DOI:** 10.1371/journal.pone.0045308

**Published:** 2012-09-17

**Authors:** Dhruva K. Mishra, Jason H. Sakamoto, Michael J. Thrall, Brandi N. Baird, Shanda H. Blackmon, Mauro Ferrari, Jonathan M. Kurie, Min P. Kim

**Affiliations:** 1 Department of Surgery, The Methodist Hospital Research Institute, Houston, Texas, United States of America; 2 Department of Nanomedicine, The Methodist Hospital Research Institute, Houston, Texas, United States of America; 3 Department of Pathology and Genomic Medicine, The Methodist Hospital, Houston, Texas, United States of America; 4 Department of Thoracic, Head and Neck Medical Oncology, The University of Texas MD Anderson Cancer Center, Houston, Texas, United States of America; 5 Department of Surgery, Weill Cornell Medical College, The Methodist Hospital, Houston, Texas, United States of America; National Center for Scientific Research Demokritos, Greece

## Abstract

We compared the growth of human lung cancer cells in an *ex vivo* three-dimensional (3D) lung model and 2D culture to determine which better mimics lung cancer growth in patients. A549 cells were grown in an *ex vivo* 3D lung model and in 2D culture for 15 days. We measured the size and formation of tumor nodules and counted the cells after 15 days. We also stained the tissue/cells for Ki-67, and Caspase-3. We measured matrix metalloproteinase (MMP) levels in the conditioned media and in blood plasma from patients with adenocarcinoma of the lung. Organized tumor nodules with intact vascular space formed in the *ex vivo* 3D lung model but not in 2D culture. Proliferation and apoptosis were greater in the *ex vivo* 3D lung model compared to the 2D culture. After 15 days, there were significantly more cells in the 2D culture than the 3D model. MMP-1, MMP-9, and MMP-10 production were significantly greater in the *ex vivo* 3D lung model. There was no production of MMP-9 in the 2D culture. The patient samples contained MMP-1, MMP-2, MMP-9, and MMP-10. The human lung cancer cells grown on *ex vivo* 3D model form perfusable nodules that grow over time. It also produced MMPs that were not produced in 2D culture but seen in human lung cancer patients. The *ex vivo* 3D lung model may more closely mimic the biology of human lung cancer development than the 2D culture.

## Introduction

Lung cancer is the most common cause of cancer-related death in the United States [1]. To date, there is no effective treatment for patients with lung cancer, and the overall 5-year survival rate has not increased significantly since 1975, 13% in 1975 and only 16% in 2005 [1]. The lack of progress in finding effective treatments for lung cancer may be due, in part, to the lack of an accurate model that mimics the biological processes that occur in patients with lung cancer. Two-dimensional (2D) petri dish cell cultures have provided great insight into the ability of tumor cells to grow, but they do not provide information about the complex interactions between the cancer cells and their environment. Animal models provide definitive tests for particular processes, but there is often a lack of correlation between expected and observed results, which may be due to the models themselves [2]. Moreover, human tumor growth and response to drug therapy in animal models do not always correlate with the findings of human trials [3]–[5]. Furthermore, animal models take several weeks to provide data about biological processes. As a result, *in vitro* 3D models have been developed over the years as an attempt to fill the gap between traditional 2D cultures and animal models.

There are currently two major types of *in vitro* 3D models. The first type takes the *in vivo* tissues of interest and explants and cultures them *in vitro*, which provides information about the short-term growth of the tissues [6], [7]. The other type grows tumor cells in a 3D artificial matrix scaffold. This *in vitro* 3D model using Matrigel has been shown to be superior to 2D culture using a petri dish for studying tumor growth [8]–[10]. The physiologic changes in the cancer cells grown on Matrigel are significantly different from those of the tumors grown in 2D culture. There are limitations, however, to the current *in vitro* 3D models. Although they provide a substrate for the tumors to grow on, the substrate is an artificial product that is not encountered by these cells in a natural setting. Moreover, these *in vitro* 3D models lack the presence of vasculature, which hinders their ability to mimic the *in vivo* environment and maintain dynamic cell behavior [5].

Here, we characterized an *ex vivo* 3D lung model that has been shown to produce growing perfusable lung nodules [11]. Unlike the *in vitro* 3D models, our *ex vivo* 3D lung model uses a natural matrix, which maintains its homology between species [12], and the decellularized matrix forms a barrier between the endothelial and epithelial spaces [13]. Thus, human lung cancer cell lines are able to form lung nodules in this *ex vivo* model with intact vasculature [11], which overcomes the limitations with *in vitro* 3D models. Moreover, the *ex vivo* 3D lung model allows the cells to grow over time, which can demonstrate a dynamic condition that is not seen in *in vitro* 3D models. We compared the growth of human lung cancer cells in this *ex vivo* 3D model with that in a 2D culture under the same culture conditions for 15 days. We found significant differences in the formation of tumor nodules, total cell numbers, proliferation rates, cell death rates, and matrix metalloproteinase (MMP) production. Moreover, the human lung cancer cells grown in the *ex vivo* 3D lung model produced MMPs that are found in human samples, whereas the cells from 2D culture did not.

## Results

### Cell Growth and Nodule Formation

**Figure 1:**
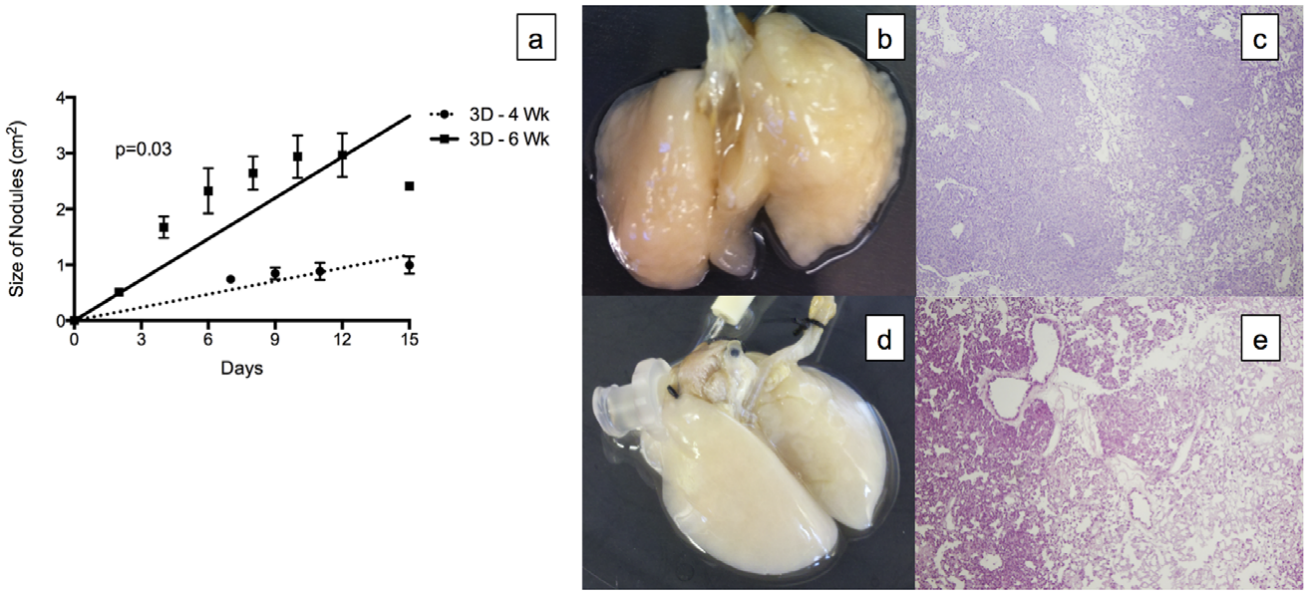
Tumor nodules formed in the *ex vivo* 3D lung model. (a) The tumor nodules increased in size over time. Significantly larger nodules formed in the decellularized lung matrix from 6-week-old rats (3D - 6 Wk, n = 2) compared to 4-week-old rats (3D - 4 Wk, n = 2, p = 0.03). The tumor nodules were denser in the 4-week-old rats (b) than in the 6-week-old rats (d). The H&E stain (4X) of the lung nodule from the 4-week-old rats showed most of the area covered by large number of cells with scant area of single layers of tumor (c) while the lung nodule from the 6- week old rats showed large areas with single layers of tumor cells (e). Error bar represents standard error of the mean.

In the *ex vivo* 3D lung model, 96.3±3.9% of A549 cells adhered to the decellularized lung matrix after 3 passes and incubation for 2 hours. The A549 lung cancer cells grown on the decellularized lung matrices from the 4-week-old (3D -4 Wk) and 6-week-old (3D -6 Wk) rats formed tumor nodules that grew over the 15-day period. Throughout the 15-day period, we were able to perfuse the 3D model with media at 6 cc per minute. The distribution of the nodules on the matrix was random, but the timing of their development was mostly uniform, with few nodules forming after day 6 for lung matrices from 4-week-old rats and day 2 for lung matricies from 6-week-old rats. Significantly larger nodules were detected on the A549 lung cancer cells grown on the decellularized lung matrices from the 6-week-old rats compared with those from the 4-week-old rats (p = 0.03, Fig. 1a). However, the nodules on the decellularized rat matrices from the 4-week-old rats were denser (Fig. 1b) than the nodules on the decellularized rat matrices from the 6-week-old rats (Fig. 1d). We calculated the density of the nodules grown on decellularized lung matrices from the 4-week-old rats and 6-week-old rats by dividing the number of the cells on day 15 divided by the total nodule size on day 15. The density of the nodules grown on the decellularized lung matrix from the 4-week-old rats (3.24±0.24 million cells/cm^2^) was significantly higher then the density of the nodules grown on the decellularized lung matrix from the 6-week-old rats (1.12±0.20 million cells/cm^2^, p = 0.02). The higher density was also evident on the H&E stain of the scaffold. The H&E stain of the lung nodule from the 4-week-old rats showed most of the area covered by large number cells with scant area of single layers of tumor (Fig. 1c) while the lung nodule from the 6-week-old rats showed large areas with single layers of tumor cells (Fig. 1e). Moreover, there is no growth of the tumor cells in the vascular space on both H&E with tumor cells growing around it without obstructing it.

In the 2D culture, the A549 cells became confluent on day 5. Over the 15-day period, there were no nodule formations.

### Structure and Cell Number

**Figure 2:**
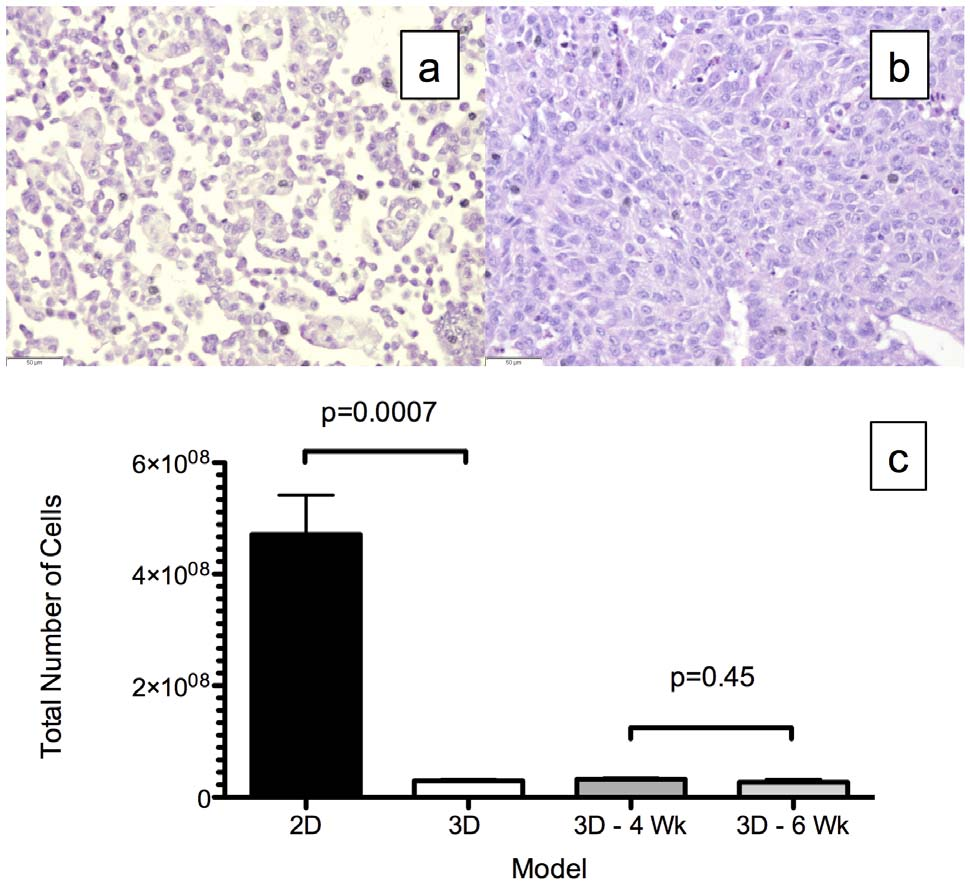
Total number of cells after 15 days. Representative Hematoxylin and eosin (H&E) staining of the cells from (a) 2D culture and (b) the *ex vivo* 3D lung model. The H&E of the *ex vivo* 3D lung model shows cell-cell and cell-matrix interactions. (c) There were significantly more cells in the 2D culture (471±70 million, n = 3) than in the *ex vivo* 3D lung model (29±3 million, n = 4, p = 0.0007). The total number of cells in the 4-week-old (32±3 million, n = 2) and 6-week-old (27±5 million, n = 2) rat matrices did not differ (p = 0.45). Error bar represents standard error of the mean.

H&E analysis of the cell block made from 2D culture showed no organization (Fig, 2a), whereas there was cell-cell and cell-matrix organization in the *ex vivo* 3D lung model, with intact vasculature (Fig. 2b). In the *ex vivo* 3D lung model, the number of A549 cells grown on the two differently sized scaffolds after 15 days did not differ significantly: 32±3 million cells from the 4-week-old rat compared to 27±5 million cells from the 6-week-old rat (p = 0.45, Fig. 2c). However, the average number of cells after 15 days in the 2D culture (471±70 million) was about 16-fold higher than the average number of cells from the *ex vivo* 3D model (29±3 million; p = 0.0007, Fig. 2c).

### Proliferation and Cell Death

**Figure 3:**
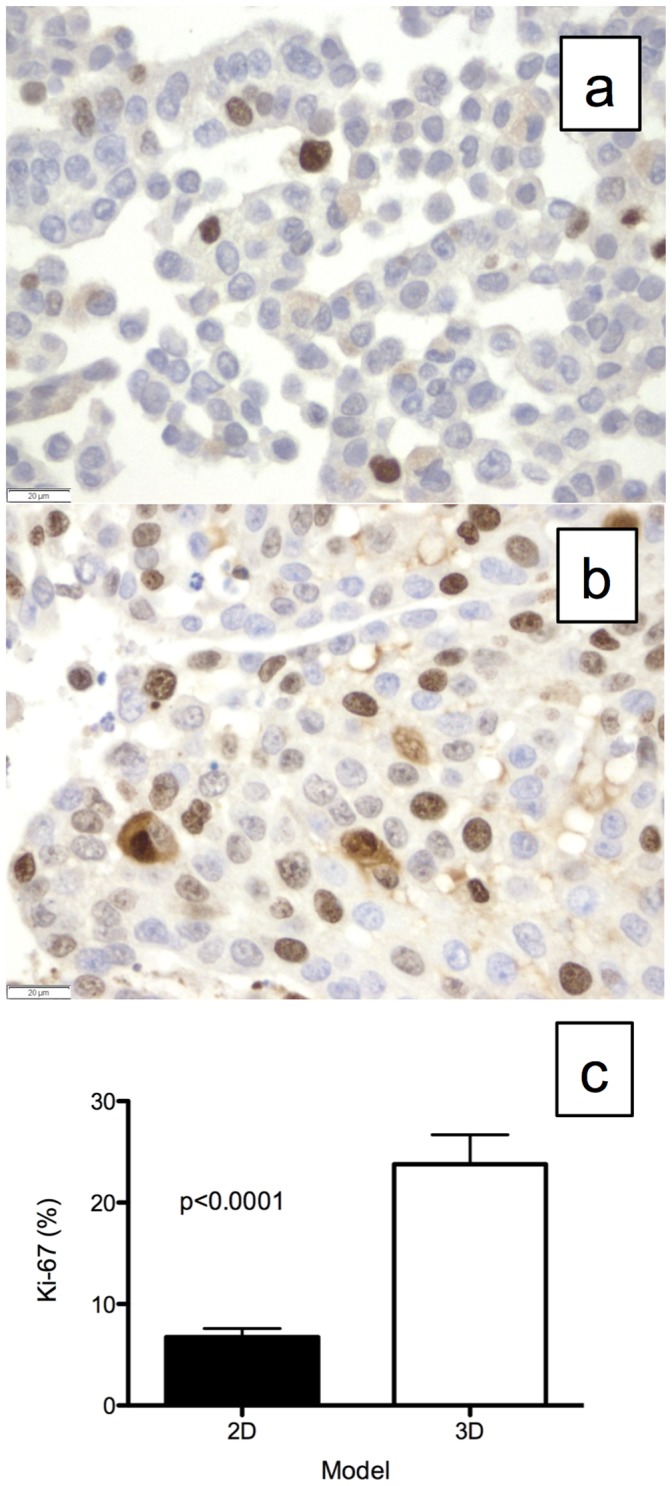
Ki-67 staining for cell proliferation index. Representative Ki-67 staining of A549 cells grown in (a) 2D culture and (b) the *ex vivo* 3D lung model. (c) There was significantly more Ki-67 staining in the *ex vivo* 3D lung model (23.8±2.9%, n = 4) compared with the 2D culture (6.7±0.9%, n = 3, p<0.0001). Error bar represents standard error of the mean.

**Figure 4:**
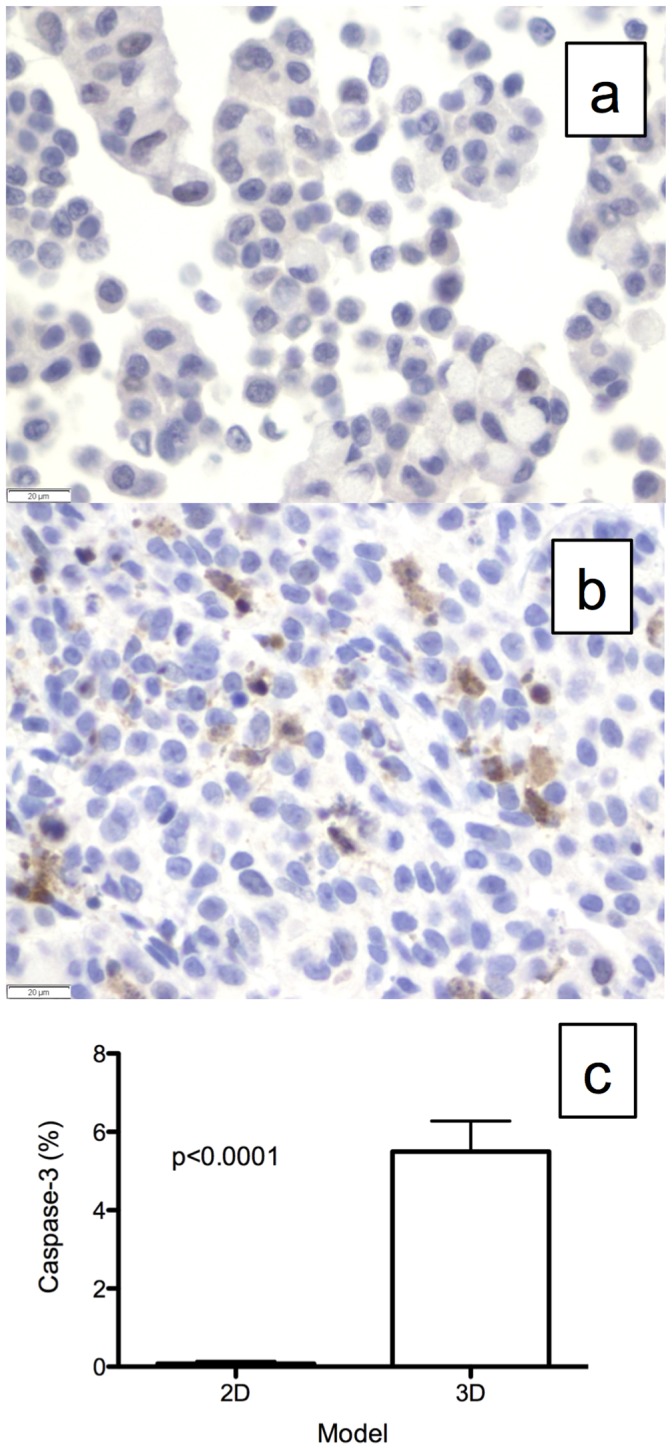
Caspase-3 staining for apoptosis or cell death. Representative Caspase-3 staining of A549 cells grown in (a) 2D culture and (b) the *ex vivo* 3D lung model. (c) There was significantly more Caspase-3 staining in the *ex vivo* 3D model (5.5±0.8%, n = 4) than in the 2D culture (0.1±0.1%, n = 3, p<0.0001). Error bar represents standard error of the mean.

The A549 cells grown in the 2D culture (Fig. 3a) and *ex vivo* 3D lung model (Fig. 3b) stained positive for Ki-67. The percentage of cells with Ki-67 staining was much higher in the *ex vivo* 3D model (23.8±2.9%) than in the 2D culture (6.7±0.9%, p<0.0001). There were very few cells in the 2D culture (Fig. 4a) that stained positive for Caspase-3, whereas there were many cells in the *ex vivo* 3D model that stained positive for Caspase-3 (Fig. 4b). A significantly higher percentage of cells in the *ex vivo* 3D model (5.5±0.8%) stained positive for Caspase-3 compared with the 2D culture (0.1±0.1%; p<0.0001, Fig. 4c).

### MMPs mRNA Level in the Models

**Figure 5:**
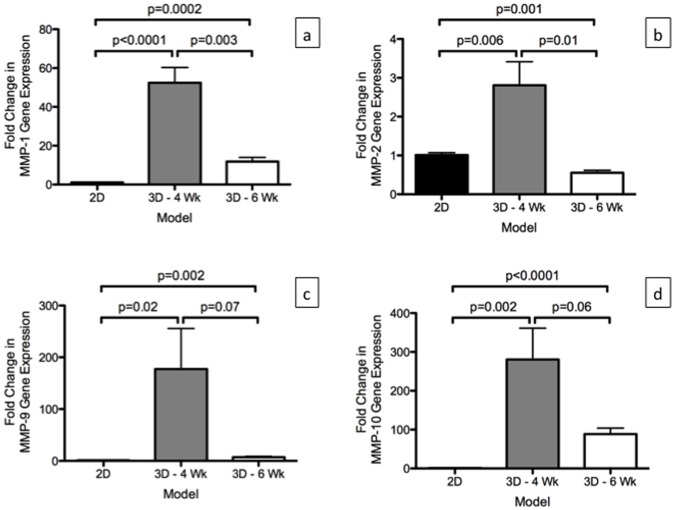
Relative mRNA expression (using 2^−(ΔΔCt)^ calculation) of MMPs in A549 cells grown in the 2D culture (2D, n = 3) and in the *ex vivo* 3D lung model on 4 week rat scaffold (3D -4 Wk, n = 2) and 6 week rat scaffold (3D -6 Wk) on day 15. There were significantly higher levels of MMP-1, MMP-9 and MMP-10 in the A549 cells in the *ex vivo* 3D models compared to the 2D culture. There was significant higher level of MMP-2 gene expression in 3D -4 Wk compared to the 2D culture however, there was significantly lower level of MMP-2 gene expression in 3D -6 Wk compared to the 2D culture. Error bar represents standard error of the mean.

There was significantly higher expression of MMP-1 (p = 0003) and MMP-2 (p = 0.01) in the A540 cells grown on the 3D -4 Wk compared to the 3D -6 Wk in the mRNA obtained from day 15 (Fig. 5a & 5b). A significantly higher expression of MMP-1 production in both 3D -4 Wk (p<0.0001) and 3D -6 Wk (p = 0.0002) was observed in comparison to the mRNA level in 2D. This was also seen with MMP-9 and MMP-10 expression (Fig. 5c & 5d). For MMP-2, there was significantly higher expression in the A549 cells grown on the 3D -4 Wk (p = 0.006) compared to the level in 2D but it was significantly lower in the A549 cells grown on the 3D -6 Wk (p = 0.001) compared to the level in 2D (Fig. 5D).

### MMPs Protein Level in the Models

**Figure 6:**
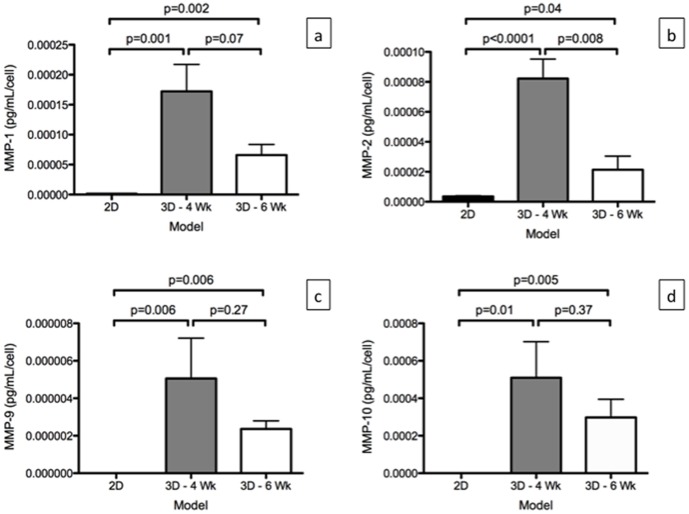
Matrix metalloproteinase (MMP) production per cell by A549 cells grown in 2D culture (n = 3) and in the *ex vivo* 3D lung model on 4 week rat scaffold (3D -4 Wk, n = 2) and 6 week rat scaffold (3D -6 Wk. n = 2). (a) There was significantly higher level of MMP-1, (b) MMP-2, (c) MMP-9, and (d) MMP-10 production per cell in the *ex vivo* 3D models compared to the 2D culture per cell. Error bar represents standard error of the mean.

MMP-1, MMP-2, MMP-9, and MMP-10 were detected in the media of A549 cells grown in the *ex vivo* 3D lung model, while only MMP-1, MMP-2, and MMP-10 were detected in the media of A549 cells grown in the 2D culture. MMP-1, MMP-2, MMP-9, and MMP-10 were not detected in the media of the *ex vivo* 3D lung model without A549 cells. Since there is significant difference in the number of cells between the 2D culture and the *ex vivo* 3D lung model at day 15, we calculated the MMP protein level per cell. We found there was significantly higher levels of MMP-1, MMP –2, MMP -9 and MMP-10 per cell in the 3D -4 Wk and 3D -6 Wk compared to the 2D culture (Fig. 6).

**Figure 7:**
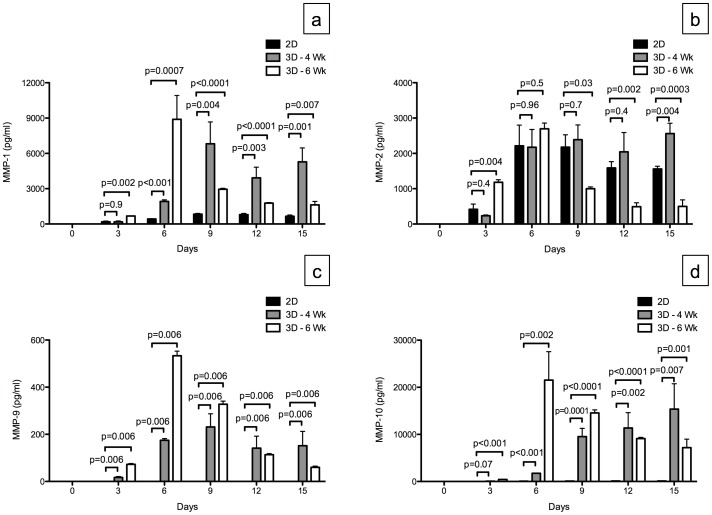
Matrix metalloproteinase (MMP) production by A549 cells grown in 2D culture (n = 3) and in the *ex vivo* 3D lung model on 4 week rat scaffold (3D -4 Wk, n = 2) and 6 week rat scaffold (3D -6 Wk, n = 2). (a) There was a significantly higher level of MMP-1 production in the *ex vivo* 3D lung models compared to the 2D culture except for 3D -4 Wk day 3 MMP-1 level which was similar to 2D level on day 3. (b) There was no consistent trend between the *ex vivo* 3D lung models and the 2D culture for MMP-2 levels among different days. (c) There was no production of MMP-9 in 2D culture and significantly higher production of MMP-9 in the *ex vivo* 3D lung model. (d) There was significantly more MMP-10 produced in the *ex vivo* 3D lung models compared to the 2D culture except for 3D -4 Wk day 3 MMP-10 level which was similar to the level on day 3 for 2D culture. Error bar represents standard error of the mean.

Next, we evaluated the MMP level on different days in the media of the 2D compared to the 3D -4 Wk and the 3D -6 Wk. The MMP levels in the *ex vivo* 3D lung model increased during the first 6 days of the experiment. Although there were more cells in the 2D culture, there were significantly higher amounts of MMP-1, MMP-9, and MMP-10 in the *ex vivo* 3D lung model compared to the 2D model for all days except for MMP-1 and MMP-10 levels on day 3 for 3D -4 Wk which was similar to the 2D model. There was no consistent relationship between the MMP-2 levels in the 2D model and the 3D -4 Wk and 3D -6 Wk models for different days (Fig. 7).

### MMPs in Patients

**Figure 8:**
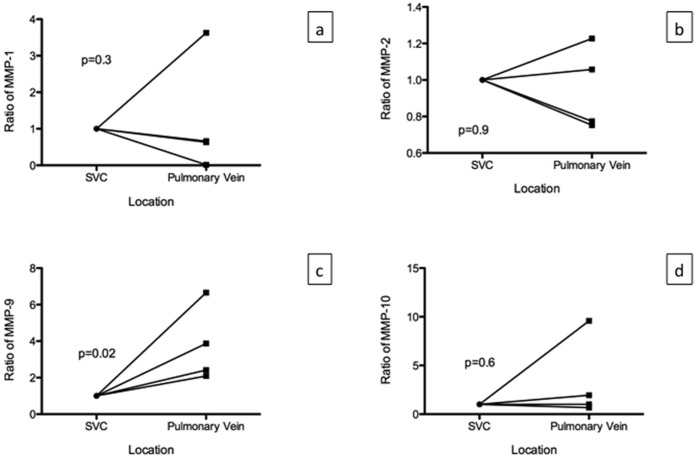
MMP levels in patients with adenocarcinoma of the lung (n = 4). MMP levels are normalized to the level found in the superior vena cava (SVC). There was no significant difference in the MMP levels between the SVC and the pulmonary vein for MMP-1 (p = 0.03), MMP-2 (p = 0.9) and MMP-10 (p = 0.6). There was significant increase in the MMP-9 levels in the pulmonary vein compared to the SVC (p = 0.02).

We analyzed samples from four patients with adenocarcinoma of the lung. None of the patients had pre-operative chemotherapy or radiation therapy. The average tumor size was 2.6±1.1 cm, and none of the patients had involvement of the lymph nodes. MMP-1, MMP-2, MMP-9, and MMP-10 were detected in the superior vena cava and pulmonary veins of the lobectomy specimens from all four patients. There was an average of 11.8±22.3 ng/mL of MMP-1, 40.4±23.2 ng/mL of MMP-2, 41.6±27.6 ng/mL of MMP-9 and 100.4±128.5 pg/mL of MMP-10 in the plasma of the blood drawn from the superior vena cava. There was an average of 7.8±13.8 ng/mL of MMP-1, 37.8±19.9 ng/mL of MMP-2, 134.5±83.3 ng/mL of MMP-9, and 105±90.9 pg/mL of MMP-10 in the plasma of the blood obtained from the pulmonary veins of the lobectomy specimens. Since there was large variation among patients for the level of MMPs, we normalized the value to the level found in the superior vena cava for each patient. We then compared the values among patients. There was no significant difference among MMP-1, MMP-2 and MMP-10 levels, however, there was significantly higher level of MMP-9 in the pulmonary vein compared to the superior vena cava (p = 0.02, Fig. 8).

## Discussion

The A549 cells grown in the *ex vivo* 3D lung model were more similar to lung cancer cells growing in patients than the A549 cells grown in 2D culture. As we have shown previously [11], the A549 cells grown in the *ex vivo* 3D lung model formed nodules, but the cells grown in 2D culture did not. The hallmark of lung cancer is the formation of a lung nodule that can be clinically detected with imaging such as a chest X-ray or computed tomography [14]. These lung cancers grow over time and metastasize to lymph nodes and other organs. The *ex vivo* 3D lung model showed nodule formation and growth over time, but the 2D culture did not. Moreover, larger nodules were detected when a larger decellularized lung matrix was used in the experiment. The likely reason for this difference is that the size of the nodule is limited by the surface area of the decellularized lung matrix. Furthermore, the A549 cells formed denser nodules on the smaller lung matrix than on the larger lung matrix, and the total number of cells after 15 days of growth on the *ex vivo* 3D lung model did not differ between the two sizes of decellularlized lung matrix. This suggests that the bigger and less dense nodules grown on the decellularized lung matrices from the 6-week-old rats had a similar number of cells as the smaller and more dense nodules grown on the matrices from the 4-week-old rats. Regardless of the size of the decellularized lung matrix used in the experiment, there was tumor nodule formation and the nodules grew over time.

There was significantly more tumor remodeling of the A549 cells grown in the *ex vivo* 3D lung model compared with those grown in the 2D culture. After 15 days, a much higher number of cells was found in the 2D culture than in the *ex vivo* 3D lung model. The growth of the cells in the 2D culture was limited by space, whereas the growth of the cells in the *ex vivo* 3D lung model seem to have been limited by factors other than space, since using a larger decellularized lung matrix did not increase the total number of the cells at the end of the experiment. In the *ex vivo* 3D lung model, the tumor cells formed nodules at certain places in the matrix, and once the nodules formed, they increased in size, with few additional nodules forming during the 15-day period. This suggests the proliferation of cells in areas of favorable growth and cell death in areas of unfavorable growth. This was evidenced by analysis of Ki-67, which is a marker of proliferation, and Caspase-3, which is a marker of cell death. There was a significantly higher amount of cell proliferation and cell death in the *ex vivo* 3D lung model compared to the 2D culture. This remodeling occurred in the context of the tumor cells being organized with cell-cell and cell-matrix interactions that are only seen in an *in vivo* setting [11].

Factors that may contribute to the formation of favorable conditions for tumor nodule growth include nutrients from the media and pockets of hypoxia created by the model [15]. The cancer cells will likely form nodules in areas that are rich with nutrients, which may allow the cells to grow better than in areas with low perfusion; thus, the cells in the nutrient-rich areas closer to blood vessels may have greater proliferation than those in areas with less perfusion. The ability of the *ex vivo* 3D lung model to mimic this condition makes it a better model of the *in vivo* environment than the 2D culture.

In addition, the *ex vivo* 3D lung model may mimic the *in vivo* environment better than *in vitro* 3D models (such as growing lung cancer cells on Matrigel) because it retains a vascular space, has the natural architecture of a perfusable organ, and most importantly, the composition of the matrix is maintained between species [12]. This two features are the most important aspect of the *ex vivo* 3D model. The intact vascular space is result of the growing the tumor cells in the acellular matrix. As the acellular matrix is created through the decellularization process, where the basement membrane is preserved and maintains the integrity of the vascular space and epithelial space. The decellularization process removes the endothelial cells from the vascular space and epithelial and mesenchymal cells from the epithelial space. The tumor cells are placed in the epithelial space through the trachea and the media is pumped through the vascular space. As the tumor grows and forms nodules, it preserves the vascular space. If the tumor destroys the vascular space then the media would not be able to pump through the scaffold at 6 cc per min. Throughout the 15-days, we did not have any problems perfusing the tumor nodule. Thus, the model creates perfusable tumor nodule. There is no other model that can deliver nutrients and remove secreted factors like this model. Moreover, unlike *in vitro* 3D models that rely on an artificial matrix, the composition of the matrix used in the *ex vivo* 3D lung model is the composition that is most likely seen by lung cancer cells in the *in vivo* environment. As cell signaling and behavior are dependent on the composition and stiffness of the extracellular matrix [9], [16], [17], the behavior of tumor cells may be more accurately represented by the *ex vivo* 3D lung model.

In addition to forming perfusable tumor nodules, the A549 cells grown in the *ex vivo* 3D lung model produced MMP-9 that is produced by lung cancer cells in patients. MMPs are a family of zinc-dependent endopeptidases that play a key role in tumor cell invasion, migration, and metastasis by degrading the extracellular matrix (ECM) and basement membranes [18]. ECM is a complex network of macromolecules such as collagen, proteoglycans, laminin, fibronectin, and many other glycoproteins. The mRNA of the MMPs were also detected on the tumor cells growing on the *ex vivo* 3D model on day 15. Moreover, the MMP-1, MMP-2, MMP-9, and MMP-10 were found in the media of the *ex vivo* 3D lung model as well as in the serum of the blood samples collected from the superior vena cava and pulmonary veins of the lobectomy lung cancer specimens. The pulmonary vein is the first major vessel encountered by secreted protein from the lung cancer, which will hold the highest concentration of the proteins secreted by the cancer. Moreover, the superior vena cava contains blood that travels to the pulmonary artery which goes to the lung with the tumor. Thus, the MMP that is likely produced by the tumor should have highest concentration in the pulmonary vein and lowest concentration in the superior vena cava. Among the four MMPs studied, we found that the MMP-9 is the only protein that met this criteria. MMP-9 was found in the media of the *ex vivo* 3D lung model while it was not found in the 2D culture. Our study confirms the previous finding that the A549 cells will not produce MMP-9 in a 2D culture [19]. MMP-9 plays an important role in the degradation of type IV collagen, which is a major structural protein for ECM and basement membranes. High serum MMP-9 levels have been shown to correlate with poor survival in patients with lung cancer [20], [21]. Thus, the presence of MMP-9 in the *ex vivo* 3D lung model suggests that it mimics *in vivo* conditions better than the 2D culture.

The strength of the current study is showing how human lung cancer cells behave in 2D compared to the *ex vivo* 3D model using the same number of cells and using the same culture media. A weakness of the current study is that we were not able to compare the *ex vivo* 3D model to the *in vitro* 3D models. We were not able to culture 25 million cells on a matrigel to provide a comparison due to the technical limitations of *in vitro* 3D models. However, we were able to show features of the *ex vivo* 3D model which were not seen in the *in vitro* 3D models such as growth of tumor nodules and preservation of the vascular space.

Overall, the human lung cancer cells grown in the *ex vivo* 3D lung model had features that mimic lung cancer growth and metastasis in patients, whereas the cells grown in 2D culture did not. The human lung cancer cells grown in the *ex vivo* 3D lung model formed perfusable tumor nodules, with secreted proteins that are important for tumor growth and metastasis. Therefore, the *ex vivo* 3D lung model may be a better model with which to study lung cancer growth and metastasis than the 2D culture and, possibly, other *in vitro* 3D models.

## Materials and Methods

We obtained informed written consent from patients to determine secretome profiles in the blood. The experiments involving patients were approved by the Institutional Review Board at the Methodist Hospital Research Institute (IRB(2) 0811-0142). All the animal experiments were carried out in accordance with all applicable laws, regulations, guidelines, and policies governing the use of laboratory animals in research. The protocols for animal experiments were approved by the Institutional Animal Care and Use Committee at the Methodist Hospital Research Institute (AUP-0910-0018).

### Patient Samples

Patients who were undergoing a lobectomy for lung cancer gave consent to have blood taken from the superior vena cava and pulmonary vein of the resected specimen. We also collected patient demographic information and the pathology results for the specimen. After patient had a central line place, we removed approximately 10 mL from the central line located in the superior vena cava in a BD Vacutainer with K_2_ EDTA (BD, Franklin Lakes, NJ, USA. Then, after the specimen was removed from the patient, the pulmonary vein was identified. We removed approximately 10 mL of blood from the vein and placed it in a BD Vacutainer with K_2_ EDTA (BD, Franklin Lakes, NJ, USA). The sample was diluted with phosphate-buffered saline (PBS) at 1∶1; then, the sample was layered on 18 mL of lymphocyte separation media and spun at 2200 *g* in a centrifuge for 20 minutes. The MMP concentration of the plasma layer was determined using a Luminex (Luminex, Austin, TX, USA) assay described below.

### Cell Culture

The human alveolar basal epithelial adenocarcinoma cell line A549 was obtained from American Type Culture Collection (Manassas, VA, USA). The cells were grown in 525 cm^2^ cell culture flasks (BD Biosciences, San Jose, CA, USA) in complete media made from RPMI 1640 medium (Hyclone, South Logan, UT, USA) supplemented with 10% fetal bovine serum (Lonza, Walkersville, MD, USA) and antibiotics (100 IU/mL penicillin, 100 µg/mL streptomycin, and 0.25 µg/mL amphotericin; MP Biomedicals, Solon, OH, USA) at 37°C in 5% CO_2_. Once cells were 85% confluent, they were washed with PBS and subjected to trypsinization using 0.25% trypsin (Cellgro, Manassas, VA, USA) to collect the cells from the flasks. The cells were washed with media and finally suspended in 30 to 50 mL of complete media.

### *Ex vivo* 3D Lung Model

We created the *ex vivo* 3D lung model as previously described [11]. We used decellularized lung matrices from 4-week-old (n = 2) and 6-week-old (n = 2) rats. The decellularized lung matrices from 4-week-old rats were significantly smaller than those from 6-week-old rats. We placed 25 million A549 cells diluted in 50 ml of complete media through the trachea of the *ex vivo* 3D lung model (n = 4). We collected the media that passively came out of the model into a 500-mL container, passed it again through the trachea three times, and incubated it at 37°C for 2 hours to allow for cell attachment. We then counted the cells in the container and determined the percentage of tumor cells seeded to the *ex vivo* 3D lung model. We placed an additional 200 mL of complete media in the bioreactor previously described [11] and allowed the media to go through the pump at 6 ml per minute and then through the oxygenator and the pulmonary artery of the *ex vivo* 3D lung model. We also ran a control *ex vivo* 3D lung model where we changed 200 ml of complete media in the bioreactor daily without placing any cells in the model. We collected the media every day from the 500-mL container and spun down the used media at 400 *g* for 5 minutes and saved 10 mL of the supernatant for secretome analysis. We replaced the used media with fresh media every day for 15 days. Every other day, we examined the *ex vivo* 3D lung model for the presence of nodules. We calculated the area per nodule and added up all of the nodule areas to obtain the total nodule size per model. After 15 days, about 5% of the *ex vivo* 3D lung model was removed and fixed in 10% formalin for hematoxylin and eosin (H&E), Ki-67, and Caspase-3 assay. We then created a single cell suspension of the rest of the lung by using mechanical breakdown followed by enzymatic degradation with collagenase and dispase at 37°C for 45 minutes. We spun down the cells at 400 *g* for 5 minutes and resuspended the pellet in 1 mL of media; the cells were then counted using the TC10 automated cell counter (Bio-Rad, Hercules, CA, USA).

### 2D Culture

We placed 25 million A549 cells in a 525-cm^2^ cell culture flask (BD) with 200 ml of complete media (n = 3). We removed the media every day and processed it in the same way as the media from the *ex vivo* 3D lung model. We saved 10 ml of the supernatant for secretome analysis. After 15 days, the cells were trypsinized and counted. We removed 5% of the cells and created a cell block on which to perform H&E, Ki67, and Caspase-3 staining.

### Histological Analysis

The lung tissues from the *ex vivo* 3D lung model and cell blocks from 2D culture were placed in 70% ethanol and analyzed in the Pathology Core Laboratory at The Methodist Hospital Research Institute. Briefly, the tissues were fixed in 10% formalin overnight, processed, and embedded in paraffin. Embedded tissues were cut into 4-micron-thick slides and dewaxed in xylene, alcohol, and water, followed by antigen retrieval with antigen-unmasking solution (Vector Laboratories, Burlingame, CA, USA) in a steamer for 20 minutes. Slides were cooled for 20 minutes at room temperature, washed in PBS, and stained with H&E [22].

For immunohistochemical analysis, the primary antibody was incubated overnight at 4°C (Ki67 and Caspase–3; Abcam, Cambridge, MA, USA), followed by the secondary antibody linked with horseradish peroxidase (Vector Laboratories) for 20 minutes. After every step, slides were washed three times with Tris-Buffered NaCl Solution with Tween 20 (Dako, Carpinteria, CA, USA). Finally, DAB (Dako) was used to develop the color for 5 minutes and the slides were rinsed in distilled water. A counterstain of modified Mayer’s Hematoxylin (Dako) was used for 20 seconds, and then the slides were rinsed sequentially with distilled water, 95% alcohol, 100% alcohol, and xylene. A permanent coverslip was mounted on the slide. Stained slides were examined by expert, board-certified pathologists, and images were captured using a microscope (Olympus, Center Valley, PA, USA). We determined the percentage of Ki-67 and Caspase-3 staining by examining five random high-power fields.

### RNA Extraction and RT-PCR

Total RNA was extracted from monolayer cell cultures as well as recellularized lung matrix with Isol-RNA Lysis reagent (5 PRIME, Gaithersburg, MD, USA) followed by SurePrep RNA cleanup and concentration kit (Fisher Scientific, Pittsburgh, PA, USA). RNA was treated with Ambion DNA-free kit (Applied Biosystems, Carlsbad, CA, USA) as per manufacturer’s instructions. cDNA was prepared using High capacity cDNA Reverse Transcription kit (Applied Biosystems) with 200 ng of total RNA and real time PCR assay was performed with SYBR green reagent (Applied Biosystem). We have used the published 18S rRNA as housekeeping genes [23]. MMP-1, MMP-2, MMP-10 [24] and MMP-9 [25] primers. A relative fold of gene expression of MMP-1, MMP-2, MMP-9 and MMP-10 was calculated using 2^−(ΔΔCt)^ formula.

### Luminex Assay

We performed a Luminex bead assay using the media collected from the *ex vivo* 3D lung models, with and without cells, and from 2D culture on days 0, 3, 6, 9, 12, and 15. We used the Millipore Milliplex Human MMP panel 2 kit (Millipore, Billerica, MA, USA), which detects active as well as inactive MMP-1, MMP-2, MMP-9, and MMP-10. In brief, all samples were taken out of storage and left at room temperature for 30 minutes. Plasma samples were diluted 20 times before use, whereas culture media were used undiluted. In a 96-well setup, 6 standards, two quality controls, one background, and 39 samples were used in duplicate. Before the samples were added, the filter plate was pre-wet with 200 µL of wash buffer and kept on the shaker for 10 minutes at room temperature. Wash buffer was removed by vacuuming, and excess buffer from the bottom was blotted with an absorbent paper towel. Assay buffer (25 µL) was added to each well and then 25 µL standard provided with the kit and samples were added to each well, followed by 25 µL of mixed beads. These were incubated for 2 hours on a shaking platform. After incubation, the fluid was gently removed by vacuuming, washed twice with wash buffer, and then incubated with 25 µL of detection antibodies for 1 hour with agitation. Following incubation, 25 µL streptavidin-phycoerythrin was added to each well and incubated for 30 minutes on plate shaker at room temperature. Finally, fluid was gently removed using a vacuum and washed twice with wash buffer, and the beads were suspended in 100 µL of sheath fluid. A Luminex 200™ instrument (Luminex, Austin, TX, USA) was used to read the plate at the following settings: gate, 8000 to 15000; events, 50 per bead); sample size, 50 µL: and timeout, 60 seconds. Results were further analyzed using MILLIPLEX Analyst Software (Millipore).

### Statistical Analysis

The differences in tumor size, number of tumor cells, percentage of Ki-67 staining, percentage of Caspase-3 staining, and MMP mRNA and protein levels between A549 lung cancer cells grown in an *ex vivo* 3D lung model compared to 2D culture were determined using Student’s t-test and Mann-Whitney test on PRISM software (GraphPad Software, La Jolla, CA, USA). A p-value <0.05 was considered significant.
